# Combined EPP and CTG Wall Approach for Distal Papilla Reconstruction in a Noncontained Intrabony Defect: A 24‐Month Follow‐Up Case Report

**DOI:** 10.1155/crid/8085709

**Published:** 2026-09-22

**Authors:** Zeynep Demir Yıldız, Guliz N. Guncu

**Affiliations:** ^1^ Department of Periodontology, Faculty of Dentistry, Hacettepe University, Ankara, Turkey, hacettepe.edu.tr

**Keywords:** case report, guided tissue regeneration, intrabony defect, periodontitis

## Abstract

**Background:**

Management of noncontained intrabony defects remains challenging because limited defect containment compromises clot stability, flap support, and regenerative outcomes. This case report describes a combined surgical approach using the entire papilla preservation (EPP) technique with connective tissue graft (CTG) wall augmentation, enamel matrix derivative (EMD), and a xenogeneic bone substitute for the management of a combined intrabony defect with distal papilla deficiency.

**Methods:**

A patient presented with a 9‐mm probing depth associated with a combined intrabony defect with a noncontained two‐wall coronal component and a three‐wall apical component. Following initial periodontal therapy, regenerative surgery was performed using the EPP technique with CTG wall augmentation, xenogeneic bone substitute, and EMD. Clinical and radiographic outcomes were evaluated over 24 months.

**Results:**

Probing depth was reduced from 9 mm at baseline to 3 mm at 24 months, accompanied by a substantial clinical attachment gain and radiographic evidence of defect fill. Distal papilla deficiency decreased from 4 to 2 mm, corresponding to an improvement from Class II to Class I, indicating partial soft‐tissue reconstruction and improved tissue stability. Radiographic evaluation demonstrated progressive defect fill and stable radiopacity throughout the follow‐up period. No postoperative complications, flap dehiscence, or biomaterial exposure were observed.

**Conclusion:**

The combined use of EPP and CTG wall with xenograft–EMD may provide favorable clinical and radiographic outcomes in challenging noncontained intrabony defects. This approach may contribute to improved soft‐tissue stability and partial reconstruction of papillary deficiencies.

## 1. Introduction

Periodontitis is characterized by bacterially induced inflammation and the breakdown of periodontal supporting tissues, leading to hard‐ and soft‐tissue deficiencies [1]. Therefore, the ultimate goal of periodontal therapy is not only to stop the progression of periodontal disease but also to regenerate the original architecture and function of the periodontal complex [2].

Periodontal regeneration is defined histologically as the regeneration of the tooth′s supporting tissues, including alveolar bone, periodontal ligament, and cementum, over a previously diseased root surface [1, 3]. Regenerative interventions is aimed at regaining lost periodontal structure in both form and function. Several approaches have been investigated for their regenerative capabilities in intrabony defects, including guided tissue regeneration (GTR), various grafting materials, enamel matrix derivative (EMD), flap operations, platelet‐rich products, and combinations of two or more techniques [4]. The biological and surgical approaches for successful regenerative interventions depend on many variables, such as defect depth and width, number of remaining bony walls, soft‐tissue deficiency, flap tension, and esthetic requirements [5, 6].

However, these techniques require high technique sensitivity, and multiple factors influence their success. Surgery‐associated factors significantly impact outcomes, in addition to patient‐related and defect‐related factors. Each step in the procedure, such as incision design, flap pattern, debridement methods, material placement, flap repositioning, and suturing, plays an important role in the outcomes. Conventional periodontal flap surgery techniques use an incision to detach the interdental papilla [5]. Although separation of the interdental papilla facilitates visualization and instrumentation of the defect area, disruption of papillary integrity may adversely affect wound stability and increase the risk of postoperative soft‐tissue complications, particularly during the early healing phase. The ideal incision and flap design should ensure primary closure of the flap and maintain space for regeneration at the interdental area [5, 7].

To promote early soft tissue healing, minimize papillary trauma, and reduce the risk of postoperative gingival recession, various papilla preservation techniques (PPT) have been proposed [8–11]. Building upon these concepts, a series of minimally invasive surgical techniques (MIST) were subsequently developed with the aim of improving wound stability, enhancing primary closure, and reducing surgical morbidity during periodontal regeneration [12, 13].

As a result, minimal invasiveness has emerged as a prevailing trend in periodontal surgery, particularly in the treatment of intrabony defects, where preservation of soft tissue integrity is critical for regenerative outcomes [5, 14]. However, despite the advantages of MIST, papillary elevation and partial disruption may still occur in deep and narrow defects, potentially compromising wound stability [5, 15]. To further address these limitations, the entire papilla preservation (EPP) technique was introduced in 2017 as a novel surgical approach designed specifically for the regenerative treatment of isolated deep intrabony defects, aiming to preserve the papilla in its entirety and optimize early wound healing [15].

In this approach, access to the defect is achieved through a buccal vertical releasing incision placed adjacent to the involved tooth, whereas the defect‐associated papilla is preserved as a single intact unit. A tunneling procedure performed beneath the papilla allows surgical access without separating the papillary tissues. This design aims to minimize surgical trauma, preserve vascularization of interdental tissues, and facilitate maintenance of primary wound closure during healing. Clinical studies evaluating the EPP technique have reported favorable early healing outcomes, low incidence of wound complications, and stable soft tissue conditions during follow‐up periods [16, 17]. Furthermore, healing at vertical releasing incision has been shown to occur without adverse events, supporting the safety and predictability of the technique [16].

The connective tissue graft (CTG) wall technique has been introduced to improve soft‐tissue management during regenerative treatment of intrabony defects, particularly in esthetically demanding cases. By increasing tissue thickness and supporting flap stability, CTG may contribute to more favorable healing and esthetic outcomes [18]. The initial CTG wall protocol combined EMD with the existing defect morphology and the CTG to support regenerative healing. However, because EMD does not provide mechanical support for space maintenance, its use alone may be inadequate in defect configurations characterized by limited containment and reduced structural support [19, 20]. The present case report describes a phenotype‐oriented regenerative strategy combining EPP with CTG wall augmentation and xenograft–EMD support in a noncontained intrabony defect configuration. This approach provides support for the blood clot and osteoconduction in regenerative tissues and prevents the collapse of the overlying CTG into bony defects.

## 2. Case Presentation

### 2.1. Clinical Presentation

A 45‐year‐old male nonsmoker patient without any systemic disease contraindicating dental treatment was referred to the clinic of periodontology with the complaint of bleeding. Furthermore, the patient reported no history of any periodontal surgeries.

**Figure 1:**
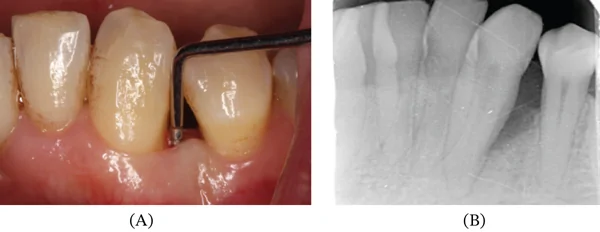
(A) PD measurement 9 mm and (B) radiographic evaluation.

**Figure 2:**
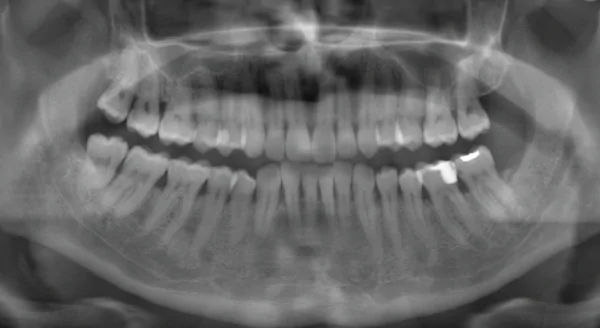
Panoramic radiograph.

Clinical evaluation demonstrated the presence of a 9–mm‐deep periodontal pocket at the distobuccal site with associated suppuration (Figure 1A). No mobility was detected. Interdental papilla loss was evident, whereas the width of keratinized tissue was adequate. On the lingual aspect of the tooth, neither recession nor deep pockets were observed. The adjacent mandibular left first premolar (#34) exhibited a gingival recession classified as Cairo Recession Type 3 (RT3), reflecting the presence of interdental attachment loss [21]. A rotated mandibular left first premolar associated with an open interdental contact was also observed. In addition, the patient′s oral hygiene measures were insufficient in this area, favoring plaque accumulation at the distal aspect of the mandibular left canine. These local plaque‐retentive factors were considered to have contributed to the site‐specific periodontal destruction. The remaining dentition exhibited no signs of periodontal attachment loss. Radiographic examination revealed a localized deep intrabony defect at the distal aspect of the mandibular left canine (#33). (Figure 1B) Full‐mouth periodontal charting together with panoramic radiographic examination demonstrated generalized mild periodontal involvement, whereas the distal aspect of Tooth #33 exhibited the most severe site‐specific destruction, with no comparable probing depth (PD) or radiographic bone loss elsewhere in the dentition (Figure 2) (Figure S1). Clinical assessment for occlusal trauma, including fremitus, tooth mobility, and wear facet evaluation, did not reveal any signs of occlusal trauma at the affected site or elsewhere in the dentition. No clinically or radiographically detectable root morphological abnormalities or anatomical defects, such as root grooves, cervical enamel projections, or enamel pearls, were identified. The patient was diagnosed with localized periodontitis, Stage III, Grade C, according to the 2017 World Workshop classification [22]. Although the patient was 45 years old, the severity of the localized destruction was disproportionate to the overall periodontal condition. A deep intrabony defect with a PD of 9 mm and suppuration was observed at a single site, whereas the remaining dentition exhibited no significant attachment loss. This site‐specific pattern suggests a history of rapid and localized disease progression rather than slow, generalized breakdown.

In the absence of systemic risk factors such as smoking or diabetes, the Grade C classification was primarily based on the extent and severity of localized tissue destruction relative to the patient′s age, indicating a high susceptibility and potentially accelerated progression at the affected site. Distal papilla deficiency (DPD) was assessed as the vertical distance from the most apical point of the interproximal contact area to the tip of the distal interdental papilla using a periodontal probe. A reduction in DPD represented a vertical gain of the interdental papillary soft tissue rather than resolution of the intrabony defect. In addition, papillary loss was classified according to the Nordland and Tarnow classification system [23], which categorizes papillary deficiency based on the relationship between the papilla tip, the contact point, and the facial cementoenamel junction (CEJ). At baseline, the defect was classified as Class II.

All clinical periodontal measurements were performed by a single experienced periodontist. Each clinical parameter was measured twice, and the mean of the two measurements was used for analysis.

### 2.2. Case Management

The patient initially underwent Steps 1 and 2 periodontal therapy according to the EFP S3‐level clinical practice guidelines. Treatment included oral hygiene instruction and motivation, professional supragingival plaque removal, and subgingival instrumentation (scaling and root planing) of the affected site following informed consent. The primary aim of initial therapy was to control inflammation and establish adequate plaque control.

Eight weeks after completion of Steps 1 and 2 periodontal therapy, the patient was re‐evaluated. Periodontal re‐evaluation included assessment of PD, clinical attachment level (CAL), bleeding on probing (BOP), and plaque control. Plaque control was satisfactory, and BOP was absent. Despite resolution of inflammation, a residual deep PD persisted. This finding was attributed to the unfavorable, noncontained morphology of the intrabony defect, which limited the potential for further improvement through nonsurgical therapy alone.

Based on these findings, the site was considered suitable for regenerative intervention. At this stage, the patient underwent Phase II periodontal treatment, which included the EPP technique combined with a CTG wall approach [15, 24].

**Figure 3:**
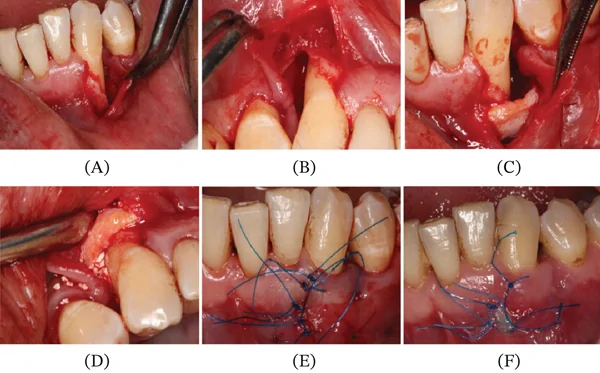
(A) Flap elevation, (B) occlusal view of the defect after degranulation, (C) CTG wall, (D) after xenograft placement, (E) suturing, and (F) after 2 weeks.

Local anesthesia was administered at the defect site through local infiltration using 4% articaine HCl with epinephrine 1:100,000, Ultracain D‐S Forte, Sanofi, Frankfurt, Germany). Following a sulcular incision around Tooth #33, a mesial vertical releasing incision was created and extended apically beyond the mucogingival junction to facilitate adequate access to intrabony defect. The flap was initially raised as a partial‐thickness dissection, extending approximately 2 mm apical to the horizontal incision, and was subsequently converted to a full‐thickness elevation beyond the mucogingival junction in a distal direction. Interdental tunnel preparation beneath the papillary tissue was accomplished using a specially designed angled tunneling instrument (Hu‐Friedy, Hu‐Friedy Mfg. Co., LLC, Chicago, Illinois, United States), allowing for atraumatic elevation of the distal interdental papilla as a full‐thickness flap (Figure 3A). Following flap elevation, a combined intrabony defect with a noncontained two‐wall coronal component and a three‐wall apical component was degranulated and root planning was performed with an ultrasonic scaler and Gracey 3/4 and 5/6 curettes (Hu‐Friedy, Hu‐Friedy Mfg. Co., LLC, Chicago, Illinois, United States) (Figure 1B). CTG, approximately 1.5 mm in thickness, was harvested from the palatal region of Teeth #25–#26 using a single‐incision technique. This approach allowed controlled harvesting of a uniformly thick graft while preserving the overlying flap, thereby providing adequate graft volume for stable adaptation and fixation to the buccal aspect of the defect. The surgical site was thoroughly irrigated with sterile saline. Subsequently, the exposed root surface was conditioned with 24% EDTA gel (Straumann Prefgel, 5 × 0.6 mL, 24% EDTA) for 2 min to remove the smear layer and was then rinsed with sterile saline. After root surface conditioning with EDTA, EMD was applied to the root surface prior to graft placement. Subsequently, CTG was positioned and sutured with 6‐0 multifilament (Doğsan, Trabzon, Türkiye) to the buccal wall of the defect to ensure close adaptation and stability (Figure 3C). After that, a bovine‐derived bone substitute (Geistlich Bio‐Oss, Geistlich Pharma AG, Wolhusen, Switzerland) was placed into intrabony defect, and additional EMD was applied (Figure 3D). The flap was sutured with 5‐0 monofilament polypropylene material (Doğsan, Trabzon, Türkiye) for optimal wound closure of the surgical area (Figure 3E).

After the surgery, patients received detailed postoperative instructions, avoiding brushing, flossing and chewing on the treated area for 2 weeks. The patient was prescribed systemic antibiotics to reduce the risk of postoperative infection, amoxicillin + clavulanic acid 1000 mg (Augmentin, GlaxoSmithKline, Türkiye) twice daily for 7 days, ibuprofen 400 mg (Brufen, Abbott Laboratories, Istanbul, Türkiye) every 8 h as needed and chlorhexidine gluconate 0.12% mouth rinse (Kloroben, Drogsan, Ankara, Türkiye) twice daily for 2 weeks. The sutures were removed 2 weeks after the surgery.

### 2.3. Treatment Outcome

The patient demonstrated good adherence to the postoperative instructions and attended all scheduled follow‐up visits. During the first year, supportive periodontal care (SPC) was provided at 3‐month intervals. During the second year, recall visits were scheduled every 6 months. At each maintenance visit, oral hygiene was reinforced, plaque control was assessed, professional supragingival plaque removal was performed when necessary, and periodontal parameters were monitored. Standardized periapical radiographs were obtained using the paralleling technique at baseline and throughout the follow‐up period. The treatment was well tolerated, and no complications requiring additional intervention were observed throughout the follow‐up period.

Postoperative healing was uneventful, except for minor gingival swelling, which resolved within 2 weeks (Figure 3F). Wound healing occurred without significant scarring or atrophy of the interdental papilla.

**Figure 4:**
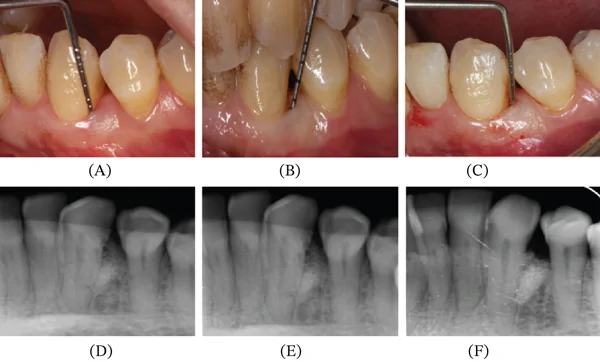
(A) At 6 months probing depth, (B) 12 months probing depth, (C) 24 months probing depth, (D) 6 months radiographic evaluation, (E) 12 months radiographic evaluation, and (F) 24 months radiographic evaluation.

At baseline, the PD was 9 mm, and interdental papilla loss at the distal aspect measured 4 mm. At 6, 12, and 24 months postoperatively, PD were maintained at clinically favorable levels, and progressive soft‐tissue stability was observed (Figures 4A–C, Table 1). At the 24‐month follow‐up, PD was reduced to 3 mm, whereas DPD decreased from 4 to 2 mm, representing a 2‐mm vertical gain in the distal interdental papillary soft tissue. This corresponded to an improvement from Nordland and Tarnow Class II to Class I. Radiographic evaluation demonstrated progressive defect fill and increased radiopacity throughout the follow‐up period (Figures 4D–F, Table 1). Residual radiopaque graft particles were still discernible at the 24‐month follow‐up, which is consistent with the well‐documented slow resorption characteristics of deproteinized bovine bone mineral and does not preclude ongoing bone remodeling and maturation.

**Table 1:** Clinical parameters.

Parameters	Baseline	6 months	9 months	12 months	24 months
FMPD	1.8	1.8	1.8	1.8	1.8
FMCAL	−1.4	−1.3	−1.3	−1.3	−1.4
PD (mm)	9	2	2	2	3
DPD (mm)	4	2	2	2	2
CAL (mm)	13	4	4	4	5

Abbreviations: CAL, clinical attachment level; DPD, distal papilla deficiency; FMCAL, full mouth clinical attachment level); FMPD, full mouth probing depth; PD probing depth.

## 3. Discussion

The present case illustrates the regenerative management of a combined intrabony defect characterized by a noncontained two‐wall coronal component and a three‐wall apical component using a phenotype‐oriented combination of the EPP technique, CTG wall augmentation, EMD, and a xenogeneic bone substitute. This combined treatment approach was selected to enhance soft‐tissue stability, improve space maintenance, and promote periodontal regeneration in a defect configuration associated with reduced natural containment and increased risk of flap collapse.

Defect morphology remains a critical determinant of regenerative outcomes [5]. Two‐walled defects typically demonstrate lower regenerative potential than three‐walled defects due to limited containment of biomaterials and clot instability. In such configurations, space‐providing strategies and meticulous soft‐tissue management become essential to optimize the conditions for regenerative healing.

The decision to incorporate the EPP technique was influenced by its well‐documented ability to preserve the integrity and vascularity of the interdental papilla, maintain primary closure, and offer a protected wound environment [15]. Evidence from clinical series indicates that EPP results in uneventful early healing with 100% primary closure during the first postoperative year, largely due to the preservation of native blood supply and minimized surgical trauma [16, 25, 26]. In this case, the complete preservation of the papilla likely contributed to the absence of wound dehiscence, flap recession, and biomaterial exposure—complications frequently reported in conventional papilla‐detaching techniques.

The CTG wall technique, originally designed to enhance root coverage and improve esthetic outcomes, also acts as a biologically stable barrier that supports space maintenance and clot stabilization over intrabony defects [18]. In line with this rationale, in the present case, CTG was employed not only for soft‐tissue augmentation but also as a strategic biological barrier to enhance clot stability and prevent graft collapse in a noncontained defect configuration. However, its application in wide, noncontained defects has been debated because of the potential for graft collapse when used with EMD alone. The addition of a particulate xenograft in this case addressed this limitation by providing a rigid, osteoconductive scaffold to support the CTG and prevent inward collapse into the defect. Similar findings have been reported in previous studies where xenograft materials paired with biologics improved defect fill and regenerative outcomes compared with biologics alone [19, 20]. The synergistic use of EMD with xenograft may also have further contributed to enhanced periodontal ligament cell activity and bone formation.

The clinical improvements observed in this case—including the reduction of PD from 9 to 3 mm and radiographic evidence of defect fill at 6 months—are consistent with outcomes of regenerative procedures in intrabony defects reported in systematic reviews [27]. In addition, DPD improved from 4 to 2 mm, corresponding to an improvement from Nordland and Tarnow Class II to Class I, indicating partial reconstruction of the interdental papilla [23].

The stable soft‐tissue architecture, absence of inflammation, and lack of recession also align with the expected benefits of minimally invasive regenerative approaches aimed at preserving soft‐tissue contours.

Residual radiopaque graft particles remained visible at the 24‐month follow‐up. Because no histologic evaluation was performed, the increased radiopacity was interpreted as representing a combination of residual Bio‐Oss particles and newly mineralized tissue rather than regenerated bone alone. This interpretation was based on serial standardized periapical radiographs demonstrating progressive reduction of the radiolucent defect and increased mineralized appearance over time. Human histologic studies have demonstrated that residual Bio‐Oss particles may persist within healing sites while becoming partially surrounded by or integrated with newly formed bone [28, 29]. Long‐term clinical and radiographic stability following the use of Bio‐Oss in periodontal intrabony defects has also been reported [30]. Therefore, the radiographic persistence of graft particles should not be interpreted as incomplete healing or unsuccessful periodontal regeneration.

Comparable regenerative approaches using the EPP technique have been increasingly reported in the literature. Ogawa et al. described a double‐sided EPP design combined with regenerative therapy, demonstrating favorable clinical attachment gain and papillary stability in intrabony defects [31]. Similarly, prospective case series evaluating EPP in conjunction with EMD and allogenic bone substitutes have shown stable clinical and radiographic outcomes over follow‐up periods extending up to 3 years [32]. Contemporary reports presenting multiple cases treated with the EPP technique further emphasize its predictability in preserving papillary integrity and minimizing postoperative recession in esthetically sensitive areas [26, 32].

More recently, the coronally advanced entire papilla preservation (CA‐EPP) flap has been introduced to enhance both buccal and interproximal soft‐tissue stability. In selected cases, incorporation of a CTG wall has been proposed to provide additional soft‐tissue support and improve space maintenance. However, most previously reported EPP‐based approaches focused primarily on flap design and regenerative biomaterials without systematically addressing phenotype‐oriented augmentation in wide, combined intrabony defects with a noncontained coronal component.

This report additionally highlights the importance of flap design and tension‐free closure in regenerative success. The combination of a vertical releasing incision with a tunnel‐based papilla approach provided adequate access while simultaneously minimizing trauma to the papillary unit. This aligns with the principles of MIST, which emphasize limited flap extension, enhanced visualization, and preservation of critical soft‐tissue components.

Although the favorable outcomes of this case are promising, certain limitations should be acknowledged. As a single‐case report, the findings cannot be generalized without caution. Additionally, the lack of histologic evaluation limits the ability to confirm true periodontal regeneration, although clinical and radiographic improvements strongly suggest successful defect resolution. Furthermore, radiographic assessment in this case was descriptive rather than quantitative, as the periapical radiographs were not acquired with a calibration reference or analyzed using standardized densitometric software; therefore, the reported radiographic improvement should be interpreted with appropriate caution. In the absence of longitudinal data and radiographic bone loss–to–age ratio assessment, the Grade C classification should be interpreted with caution, as it relies primarily on the severity and site‐specific nature of localized tissue destruction relative to the patient′s age. Nevertheless, the present case suggests that this treatment approach may be considered as a feasible option in selected cases with similar defect morphology. However, further controlled clinical studies are required before any conclusions can be drawn regarding its comparative effectiveness or superiority over other regenerative techniques.

## 4. Conclusion

Within the limitations of a single‐case report, the integration of the EPP technique with CTG wall augmentation and a xenograft–EMD combination resulted in favorable clinical and radiographic outcomes over a 24‐month period in a challenging, noncontained intrabony defect. This biologically and surgically synergistic, phenotype‐oriented approach appears to enhance soft‐tissue stability, biomaterial containment, and long‐term periodontal healing. Further controlled clinical studies are warranted to confirm the effectiveness and reproducibility of this combined regenerative strategy.

## Author Contributions

Zeynep Demir Yıldız: conceptualization (equal), formal analysis (lead), methodology (lead), project administration (equal), visualization (equal), writing—original draft preparation (lead), data curation (lead), and writing—review and editing (equal). Guliz N. Guncu: conceptualization (equal), supervision (lead), project administration (equal), and writing—review and editing (lead).

## Funding

No funding was received for this manuscript.

## Ethics Statement

Written informed consent was obtained from the patient for publication of this case report and the accompanying clinical and radiographic images. Ethical approval was not required for this single case report in accordance with institutional policy.

## Conflicts of Interest

The authors declare no conflicts of interest.

## Supporting Information

Additional supporting information can be found online in the Supporting Information section.

## Supporting information


**Supporting Information 1** Figure S1: Full‐mouth periodontal chart at the initial examination, showing the periodontal probing depths and clinical attachment levels.

**Supporting Information 2**  

## Data Availability

The data that support the findings of this study are available on request from the corresponding author. The data are not publicly available due to privacy or ethical restrictions.
