# Supplementary material for: Combined EPP and CTG Wall Approach for Distal Papilla Reconstruction in a Noncontained Intrabony Defect: A 24‐Month Follow‐Up Case Report
Source: Case Rep Dent. 2026 Sep 22;2026:8085709. doi: 10.1155/crid/8085709 (PMC13598427; doi:10.1155/crid/8085709)
Supplement: Supplementary file 1 [file CRID-2026-8085709-s001.pdf]

CARE Checklist of information to include when writing a case report

CC BY-NC-ND

Topic	Item	Checklist item description	Reported on Line
Title	1	The diagnosis or intervention of primary focus followed by the words "case report"	Title Page 1-2
Key Words	2	2 to 5 key words that identify diagnoses or interventions in this case report, including "case report"	58
Abstract (no references)	3a	Introduction: What is unique about this case and what does it add to the scientific literature?	38-40
	3b	Main symptoms and/or important clinical findings	42
	3c	The main diagnoses, therapeutic interventions, and outcomes	43-53
	3d	Conclusion—What is the main "take-away" lesson(s) from this case?	54-57
Introduction	4	One or two paragraphs summarizing why this case is unique (**may include** references)	138-142
Patient Information	5a	De-identified patient specific information	146-149
	5b	Primary concerns and symptoms of the patient	150-151
	5c	Medical, family, and psycho-social history including relevant genetic information	N/A
	5d	Relevant past interventions with outcomes	148-149
Clinical Findings	6	Describe significant physical examination (PE) and important clinical findings	151-156
Timeline	7	Historical and current information from this episode of care organized as a timeline	N/A
Diagnostic Assessment	8a	Diagnostic testing (such as PE, laboratory testing, imaging, surveys)	155
	8b	Diagnostic challenges (such as access to testing, financial, or cultural)	N/A
	8c	Diagnosis (including other diagnoses considered)	157-170
	8d	Prognosis (such as staging in oncology) where applicable	N/A
Therapeutic Intervention	9a	Types of therapeutic intervention (such as pharmacologic, surgical, preventive, self-care)	174-215
	9b	Administration of therapeutic intervention (such as dosage, strength, duration)	219-226
	9c	Changes in therapeutic intervention (with rationale)	N/A
Follow-up and Outcomes	10a	Clinician and patient-assessed outcomes (if available)	233-240
	10b	Important follow-up diagnostic and other test results	Table 1
	10c	Intervention adherence and tolerability (How was this assessed?)	229-232
	10d	Adverse and unanticipated events	230-231
Discussion	11a	A scientific discussion of the strengths AND limitations associated with this case report	310-313
	11b	Discussion of the relevant medical literature **with references**	290-303
	11c	The scientific rationale for any conclusions (including assessment of possible causes)	304-317
	11d	The primary "take-away" lessons of this case report (without references) in a one paragraph conclusion	320-326
Patient Perspective	12	The patient should share their perspective in one to two paragraphs on the treatment(s) they received	N/A
Informed Consent	13	Did the patient give informed consent? Please provide if requested	Yes ☐ No ☐
