# Supplementary material for: Combined EPP and CTG Wall Approach for Distal Papilla Reconstruction in a Noncontained Intrabony Defect: A 24‐Month Follow‐Up Case Report
Source: Case Rep Dent. 2026 Sep 22;2026:8085709. doi: 10.1155/crid/8085709 (PMC13598427; doi:10.1155/crid/8085709)
Supplement: Supplementary file 2 [file CRID-2026-8085709-s002.pdf]

PERIODONTAL CHART

Patient ID: P-Nummer | Date: 01.11.2023

Patient Last Name: | First Name: | Date of Birth: 13.08.1969

☑ Initial Examination ☐ Re-evaluation | Clinician:

	18	17	16	15	14	13	12	11	21	22	23	24	25	26	27	28
Mobility	0	0	0	0	0	0	0	0	0	0	0	0	0	0	0	
Implant																
Furcation																
Bleeding on Probing																
Plaque																
Gingival Margin	0 0 0	0 0 0	0 0 1	1 1 1	1 1 1	1 1 1	1 1 1	1 0 1	1 0 1	1 0 1	1 0 1	1 0 1	1 0 1	1 0 0	0 1 1	
Probing Depth	3 2 3	3 2 3	3 1 3	3 1 2	2 1 2	2 1 2	2 1 2	2 1 2	2 1 2	2 1 2	2 1 2	2 2 2	2 2 2	3 1 3	3 1 3	
Attachment Level	3 2 3	3 2 3	3 1 2	2 0 1	1 0 1	1 0 1	1 0 1	1 1 1	1 1 1	1 1 1	1 1 1	1 2 1	1 2 1	2 1 3	3 0 2	

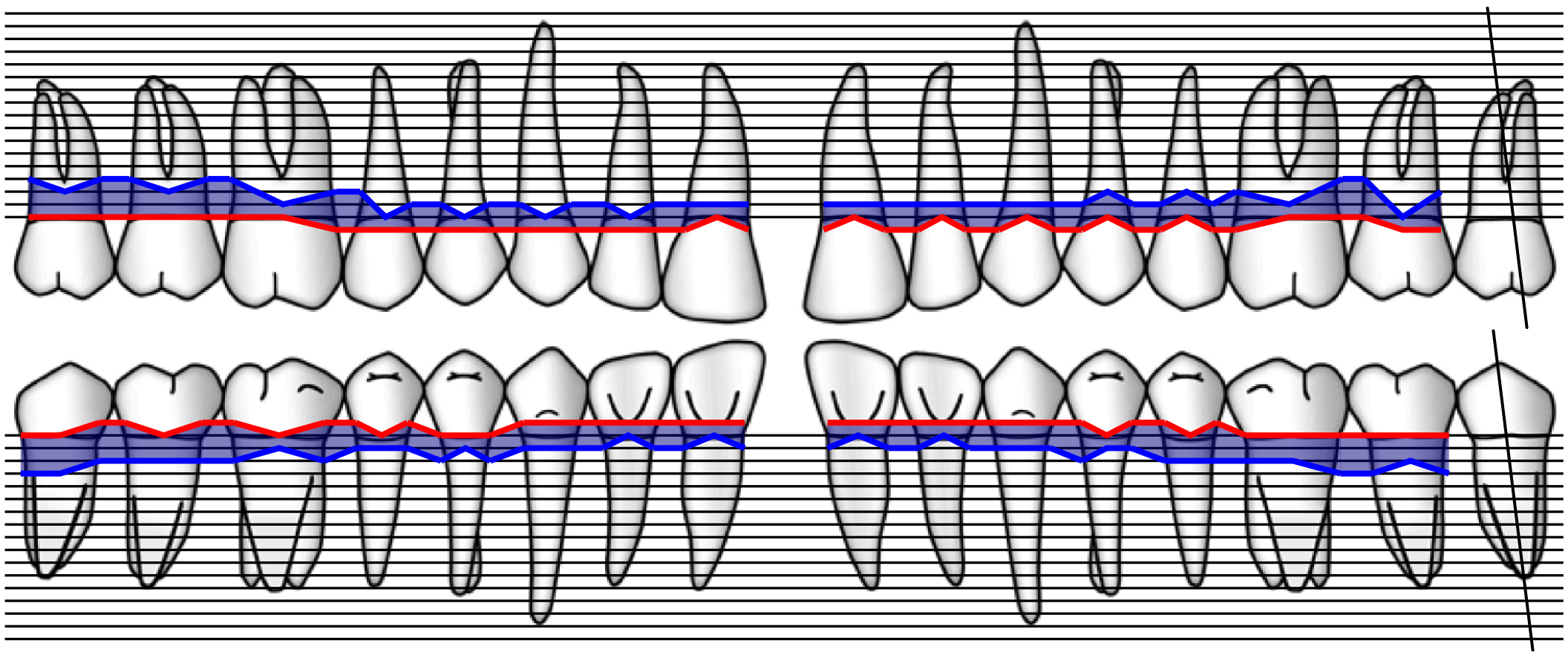

	18	17	16	15	14	13	12	11	21	22	23	24	25	26	27	28
Gingival Margin	0 0 1	1 0 1	1 0 1	1 0 1	0 0 0	1 1 1	1 1 1	1 1 1	1 1 1	1 1 1	1 1 1	1 0 1	1 0 1	0 0 0	0 0 0	
Probing Depth	3 3 3	3 2 3	3 1 3	2 1 2	2 1 2	2 2 2	2 1 2	2 1 2	2 1 2	2 1 2	2 2 2	3 1 2	3 2 3	2 2 3	3 2 3	
Attachment Level	3 3 2	2 2 2	2 1 2	1 1 1	2 1 2	1 1 1	1 0 1	1 0 1	1 0 1	1 0 1	1 1 1	2 1 1	2 2 2	2 2 3	3 2 3	
Plaque																
Bleeding on Probing																
Furcation																
Note																

Avg. Probing Depth: **1.8 mm** Avg. Attachment Level: **1.4 mm** BOP: **7%** PI: **12%**

	48	47	46	45	44	43	42	41	31	32	33	34	35	36	37	38
Note																
Furcation																
Bleeding on Probing																
Plaque																
Gingival Margin	0 0 0	0 0 0	0 0 0	1 0 1	1 0 1	1 0 0	0 -1 0	0 -2 0	0 -2 0	0 -1 1	2 2 0	0 0 1	1 0 1	1 0 1	1 0 0	
Probing Depth	0 0 0	0 0 0	0 0 0	3 2 3	2 2 2	2 1 1	1 1 1	1 1 1	1 1 1	1 1 2	3 2 2	0 0 0	0 0 0	0 0 0	0 0 0	
Attachment Level	0 0 0	0 0 0	0 0 0	2 2 2	1 2 1	1 1 1	1 2 1	1 3 1	1 3 1	1 2 1	1 0 2	0 0 -1	-1 0 -1	-1 0 -1	-1 0 0	

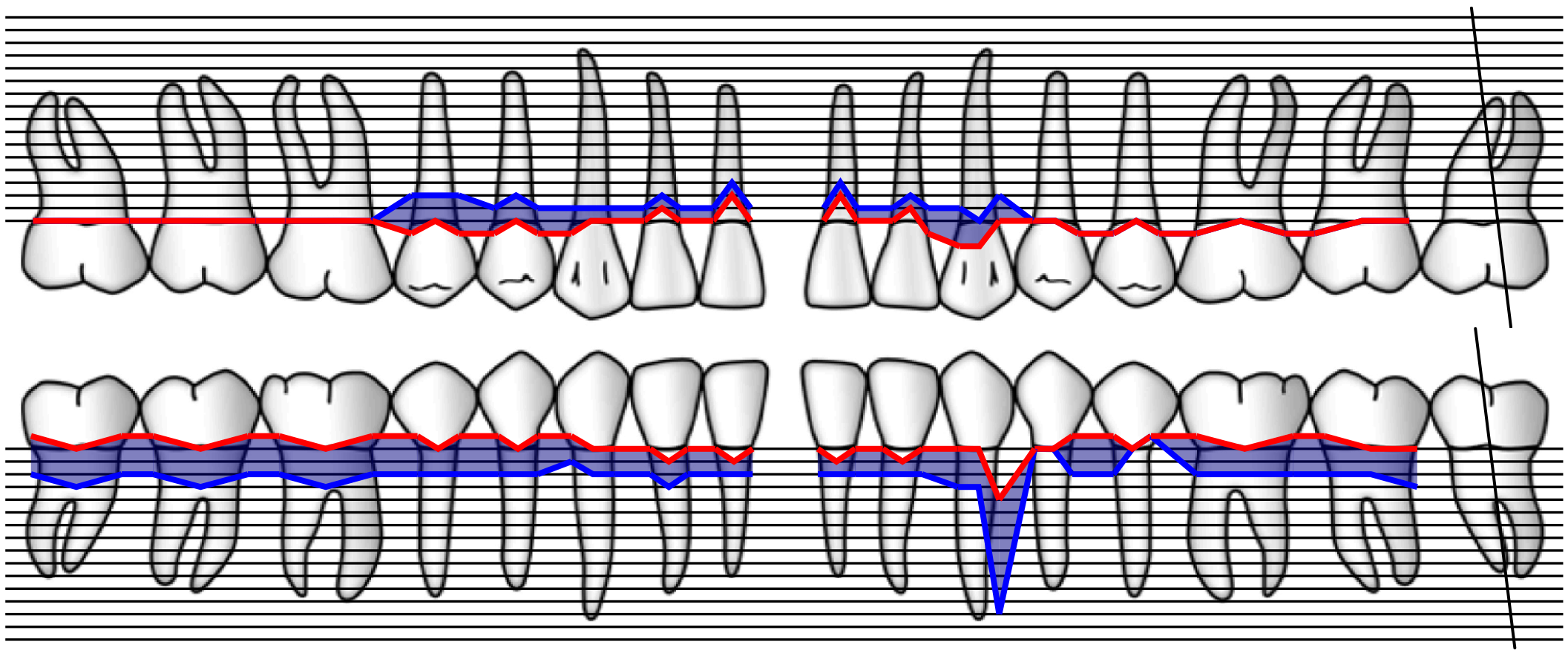

	48	47	46	45	44	43	42	41	31	32	33	34	35	36	37	38
Gingival Margin	1 0 1	1 0 1	1 0 1	1 0 1	1 0 1	1 0 0	0 -1 0	0 -1 0	0 -1 0	0 -1 0	0 0 -4	0 0 1	1 0 1	1 0 1	1 0 0	
Probing Depth	3 3 3	3 3 3	3 3 3	3 2 3	3 2 3	2 2 2	2 2 2	2 1 2	2 1 2	2 1 2	3 3 9	0 0 3	3 0 0	3 2 3	3 2 3	
Attachment Level	2 3 2	2 3 2	2 3 2	2 2 2	2 2 2	1 2 2	2 3 2	2 2 2	2 2 2	2 2 2	3 3 13	0 0 2	2 0 -1	2 2 2	2 2 3	
Plaque																
Bleeding on Probing																
Furcation																
Implant																
Mobility	0	0	0	0	0	0	0	0	0	0	0	0	0	0	0	

www.periodontalchart-online.com

Copyright © 2026 by www.perio-tools.com
